# Frozen Shoulder as a Systemic Immunometabolic Disorder: The Roles of Estrogen, Thyroid Dysfunction, Endothelial Health, Lifestyle, and Clinical Implications

**DOI:** 10.3390/jcm14207315

**Published:** 2025-10-16

**Authors:** Santiago Navarro-Ledesma

**Affiliations:** Department of Physiotherapy, Faculty of Health Sciences, Campus of Melilla, University of Granada, Querol Street 5, 52004 Melilla, Spain; snl@ugr.es

**Keywords:** frozen shoulder, lifestyle, inflammation, metabolic factors, pain, estrogen, thyroid, endothelial

## Abstract

Frozen shoulder (FS), traditionally regarded as an idiopathic musculoskeletal disorder characterized by pain, stiffness, and capsular fibrosis, is increasingly recognized as the clinical manifestation of systemic endocrine, metabolic, vascular, and immunological dysfunctions. This narrative review reframes FS within a broader neuro–endocrine–immunometabolic model, emphasizing the central role of estrogen deficiency, resistance, and receptor-level disruption, together with their interactions with thyroid dysfunction, endothelial health, and lifestyle-related low-grade inflammation (LGI). Evidence from epidemiological, clinical, and mechanistic studies shows that estrogen signaling failure weakens anti-inflammatory, antifibrotic, and antioxidant defenses, predisposing peri- and postmenopausal women to more severe FS phenotypes. Thyroid dysfunction, particularly hypothyroidism, further contributes to fibrosis and pain sensitization. Endothelial dysfunction—driven by poor diet, advanced glycation end-products (AGEs), and oxidative stress—impairs vascular integrity and promotes local microvascular inflammation. In parallel, lifestyle factors such as sedentarism, circadian misalignment, psychosocial stress, and environmental exposures sustain systemic LGI and hormonal resistance. Together, these interconnected mechanisms suggest that FS is not merely a localized joint pathology but a systemic disorder requiring integrative clinical strategies that combine orthopedic management with endocrine evaluation, metabolic monitoring, dietary interventions, circadian health, and stress regulation. In addition, this review outlines specific clinical implications, highlighting how an integrative, personalized approach that targets hormonal, metabolic, vascular, and lifestyle dimensions may improve pain, function, and long-term prognosis in FS. This paradigm shift underscores the need for future research to focus on stratified patient profiling and interventional trials targeting hormonal, vascular, and lifestyle axes to improve outcomes, particularly in women who remain disproportionately affected by FS.

## 1. Introduction

Frozen Shoulder (FS), also referred to as adhesive capsulitis, is a chronic and often debilitating musculoskeletal condition characterized by progressive pain, stiffness, and restricted range of motion in the glenohumeral joint [1,2]. It primarily affects the capsular structures of the shoulder, leading to capsular thickening, fibrosis, and chronic synovial inflammation [3,4]. Despite being a relatively common clinical entity, its etiopathogenesis remains poorly understood, and current treatment strategies are frequently limited to symptom management rather than targeting underlying mechanisms [4,5]. Epidemiological studies report a prevalence of 2–5% in the general population, which increases significantly in individuals with comorbidities such as diabetes mellitus, thyroid disorders, and autoimmune diseases [6,7,8,9,10]. Importantly, FS demonstrates a notable sex dimorphism, with a higher prevalence in women, particularly between the ages of 40 and 60, and a more prolonged and symptomatic course compared to men [11]. This sex-based disparity remains largely underexplored in clinical and mechanistic research [12,13].

Over time, FS has been referred to using various terms—adhesive capsulitis, primary idiopathic frozen shoulder, or simply FS—depending on the clinical context and presumed etiology [2]. While secondary FS may occur after trauma, surgery, or prolonged immobilization, primary FS appears spontaneously, often without a clear inciting factor, further complicating diagnosis and treatment planning [1,2]. Traditionally, FS has been considered a local mechanical and inflammatory disorder. However, emerging evidence supports a broader and more systemic conceptualization, implicating chronic low-grade inflammation (LGI), neuroimmune dysregulation, and metabolic dysfunction in its pathogenesis [5,8,14]. These perspectives open the door to integrative models that account for the influence of endocrine, nutritional, and psychosocial factors, particularly those related to female hormonal physiology.

Given the predominance of FS in women, especially around perimenopause and menopause—a period marked by a sharp decline in estrogen levels—the female sex hormonal axis emerges as a promising and under-investigated component in the etiological framework of FS [15]. Estrogens are known to exert anti-inflammatory, neuroprotective, and immunomodulatory effects, and their decline may create a systemic environment that favors fibrotic, inflammatory, and pain-sensitized states [16,17,18]. This hormonal transition may intersect with other metabolic and lifestyle factors (e.g., diet, stress, physical inactivity), contributing to the onset and progression of FS [14].

In this narrative review, our aim is to reframe FS as a systemic disorder rather than an isolated musculoskeletal condition. We explore the interconnected roles of the female sex hormonal axis—particularly estrogen deficiency, resistance, and receptor-level interference—alongside thyroid dysfunction, endothelial dysregulation, and lifestyle-driven low-grade inflammation. By integrating evidence from endocrinology, immunometabolism, vascular biology, and psychoneuroimmunology, we propose a comprehensive framework in which FS emerges as the clinical expression of hormonal, metabolic, and environmental imbalances. This perspective not only clarifies the marked female predominance and metabolic associations of FS but also highlights actionable targets for diagnosis, prognosis, and treatment. Ultimately, this review emphasizes the need for integrative and personalized strategies in physiotherapy and clinical care, bridging orthopedic, metabolic, and endocrine dimensions to improve outcomes in patients with FS.

## 2. Methods

This review was conducted as a narrative–scoping review aimed at synthesizing multidisciplinary evidence that reframes FS as a systemic, immuno-metabolic, endocrine-modulated disorder. The approach prioritized conceptual completeness and mechanistic triangulation across domains (endocrine, metabolic, vascular, neuroimmune, lifestyle) rather than exhaustive enumeration of all studies, consistent with scoping methodology, thus a PRISMA numerical flow diagram was not carried out. While not a systematic review, we prospectively defined the mechanistic domains, core questions, data sources, eligibility criteria, and an evidence-grading scheme to enhance transparency and reproducibility. Where applicable, reporting was informed by PRISMA-ScR guidance (without claiming full systematic compliance).

### 2.1. Sources and Time Frame

We searched the following databases: MEDLINE/PubMed, Embase, Scopus, Web of Science Core Collection, and the Cochrane Library. Searches covered January 2000 to May 2025, with English or Spanish language restrictions. Reference lists of key reviews and guidelines were back-snowballed to identify additional primary sources. Preprints were not included unless subsequently published in peer-reviewed journals. A full, domain-stratified search matrix (databases, exemplar queries, inclusions/exclusions) is provided in Supplementary Materials.

### 2.2. Eligibility Criteria

#### 2.2.1. Inclusion

Clinical human studies (epidemiology, cross-sectional, cohort, case–control, RCTs) on FS and: sex hormones/estrogens, thyroid axis, metabolic biomarkers (HbA1c, lipids, AGEs), endothelial function/NO/ADMA, microbiome, sleep/circadian health, psychoneuroimmunology, lifestyle/exposome, and clinical interventions.

Mechanistic/experimental studies (in vitro, animal) when directly informative of pathways relevant to FS (e.g., estrogen receptor signaling, TGF-β/ECM remodeling, AGE-RAGE, endothelial biology), used to support biological plausibility.

High-quality reviews/consensus statements (narrative/systematic/metaanalysis) to contextualize domains and summarize clinical signals.

#### 2.2.2. Exclusion

Non-FS shoulder conditions without explicit FS subgroup analysis; conference abstracts without full text; letters/editorials without primary data; non-English/Spanish full texts; preprints not peer-reviewed; duplicated cohorts; pediatric populations; pure surgical technique papers without pathophysiological content. Studies whose primary focus was unrelated to the predefined mechanistic domains were also excluded.

### 2.3. Study Selection and Data Handling

Given the single-author nature of this review, screening and selection were performed by the author using the pre-specified domains/criteria. To mitigate selection bias, the process followed a two-pass workflow: (1) title/abstract screen against domain questions; (2) full-text evaluation for eligibility and domain mapping. A screening log (retrieved, included, excluded with main reasons) was maintained. Data were extracted into structured evidence tables by domain (clinical vs. mechanistic signals) capturing design, population, exposure/biomarker, outcome, and level of evidence. Each record was explicitly tagged as Clinical (human), Translational (human tissue/biomarker), or Experimental (animal/in vitro). Clinical evidence was preferentially used for practice-oriented statements; experimental findings were used to explain mechanisms and are labeled to avoid over-extrapolation. Where evidence originated from animal/in vitro models, this is stated in text and in the tables.

### 2.4. Evidence Appraisal and Synthesis

We did not perform formal risk-of-bias scoring, consistent with scoping aims. Instead, we graded contributions as High (meta-analyses/RCTs or large human cohorts), Moderate (observational human studies or convergent multi-study signals), or Low (mechanistic/experimental or expert consensus). Narrative synthesis integrated human clinical signals with biological plausibility from experimental data, making explicit where inferences remain hypothesis-generating. Sections and summary tables clearly separate clinical versus experimental evidence lines. To strengthen interpretability, we distinguish throughout between established clinical associations, translational correlates, and emerging mechanistic hypotheses; recommendations tied primarily to mechanistic or consensus sources are identified as such. No ethics approval was required (secondary synthesis of published data); no protocol was registered given the narrative–scoping design.

## 3. Current Knowledge and Latest Hypotheses on Frozen Shoulder Pathophysiology

Traditionally, the pathophysiology of FS has been understood through the lens of localized inflammation, capsular fibrosis, and contracture of the glenohumeral joint, often associated with minor trauma, immobilization, or idiopathic onset [4,19]. Histologically, FS is characterized by chronic synovial inflammation, fibroblastic proliferation, and excessive collagen deposition, particularly in the rotator interval and coracohumeral ligament [20,21]. However, this conventional view has proven insufficient to explain several clinical observations—such as the prolonged and variable course, the incomplete recovery in many patients, and the clear female predominance, especially in peri- and postmenopausal women. Consequently, emerging evidence has led to a more integrative and systemic perspective on the etiology of FS [5,8,14].

Recent studies suggest that FS may be a manifestation of a broader systemic LGI and immunometabolic dysfunction, sharing mechanistic pathways with other fibrosing and pain-related disorders [14]. Elevated levels of pro-inflammatory cytokines such as IL-1β, TNF-α, and IL-6 have been observed in the synovial fluid and capsular tissue of FS patients, indicating an active immune-inflammatory component in the early and intermediate stages of the disease [22]. Additionally, FS has been strongly associated with systemic comorbidities including type 2 diabetes mellitus, dyslipidemia, thyroid dysfunction, and metabolic syndrome [9,23]. These conditions are known to promote oxidative stress, microvascular damage, and tissue fibrosis via AGEs, leptin resistance, and chronic activation of the HPA axis [5,24,25,26,27,28]. These systemic factors may interact with local joint environments, perpetuating inflammation and altering fibroblast behavior toward a profibrotic phenotype. Furthermore, chronic psychological stress, depression, and sleep disorders—conditions more prevalent in FS patients—have been implicated in the amplification of pain perception and central sensitization [3,29,30]. The chronic activation of stress-related neuroendocrine axes (particularly HPA and the autonomic nervous system) can exacerbate systemic inflammation and pain processing, linking FS to biopsychosocial models of chronic musculoskeletal conditions [5].

In this evolving framework, the female sex hormonal axis, particularly estrogen signaling, is gaining attention as a potentially crucial modulator of FS onset, severity, and prognosis [15]. The notably higher prevalence of FS in women, especially around menopause, has prompted hypotheses about the protective and regulatory roles of estrogens on joint inflammation, connective tissue integrity, and pain modulation [31]. These hormonal changes could act synergistically with metabolic and inflammatory imbalances, making postmenopausal women particularly vulnerable [15].

Therefore, the pathophysiology of FS may be better understood as a multifactorial condition, where hormonal, immunometabolic, psychosocial, and environmental factors converge to disrupt joint homeostasis, trigger chronic inflammation, and promote fibrotic remodeling. This integrative model paves the way for new therapeutic strategies that go beyond localized treatment and target systemic pathways involved in FS progression.

## 4. The Female Sex Hormonal Axis: Neuroendocrine, Immunologic, and Metabolic Dimensions

The female sex hormonal axis, primarily governed by estrogens, progesterone, and their regulatory interplay with the hypothalamic-pituitary-gonadal (HPG) axis, plays a critical role not only in reproductive physiology but also in systemic homeostasis—including immune function, pain perception, inflammatory control, and tissue remodeling [32,33]. In recent years, the understanding of this axis has expanded beyond its reproductive role, unveiling complex neuroendocrine and immunometabolic mechanisms that may be especially relevant in conditions such as FS [34,35].

### 4.1. Neuroendocrine Influence

Estrogens, particularly 17β-estradiol, exert significant effects on the central and peripheral nervous systems, modulating neurotransmitter release, neuronal plasticity, and pain signaling. Estrogen receptors (ERα and ERβ) are widely expressed in the brain, dorsal root ganglia, and spinal cord, where they regulate nociceptive pathways, stress responses, and mood [34,36]. Declining estrogen levels during menopause have been associated with increased central sensitization, decreased pain thresholds, and heightened responsiveness to inflammatory stimuli—factors that may exacerbate the chronic pain and stiffness observed in FS [37,38]. Moreover, estrogens interact with the HPA axis, modulating cortisol secretion and stress resilience [39,40]. In estrogen-deficient states, this axis may become dysregulated, contributing to pro-inflammatory neuroendocrine activity [41]. Chronic stress, in turn, can lower circulating estrogen levels, perpetuating a vicious cycle of neuroendocrine imbalance [40].

### 4.2. Immunologic Modulation

From an immunological perspective, estrogens possess biphasic effects: at low concentrations, they tend to promote pro-inflammatory activity, while at physiological or replacement levels, they often exert anti-inflammatory effects [42]. Estrogens enhance the function of regulatory T cells (Tregs), suppress pro-inflammatory macrophage phenotypes (M1), and reduce the expression of cytokines such as IL-1β, IL-6, and TNF-α—all of which are implicated in the pathogenesis of FS [43,44]. Additionally, estrogens stabilize mast cells and inhibit NF-κB signaling, a central inflammatory pathway activated by dietary and metabolic stressors [45]. This anti-inflammatory potential becomes particularly relevant in the context of low-grade systemic LGI, a common feature in FS patients with metabolic comorbidities [5]. Therefore, estrogen decline may shift the immune environment toward a pro-inflammatory and profibrotic state, favoring the activation of fibroblasts and excessive collagen production in the shoulder capsule [15,34].

### 4.3. Metabolic Regulation

Estrogens also play a crucial role in metabolic homeostasis, influencing lipid metabolism, glucose regulation, mitochondrial function, and oxidative stress resistance [46,47]. They promote insulin sensitivity, reduce visceral fat accumulation, and modulate adipokine release (e.g., leptin and adiponectin), all of which are known to impact systemic inflammation and joint health [48,49]. The decline in estrogen levels during menopause is associated with increased risk of metabolic syndrome, type 2 diabetes, and dyslipidemia, conditions highly prevalent in FS patients [7,9,19,50,51]. These metabolic alterations can create a systemic environment conducive to oxidative stress and vascular dysfunction, which in turn may impair tissue repair mechanisms and contribute to fibrosis [26,27,52].

One important connection lies in the role of cholesterol, both as a cardiovascular risk factor and a precursor to estrogen synthesis [53,54]. Cholesterol is converted into pregnenolone in mitochondria by the enzyme cytochrome P450scc (CYP11A1), a rate-limiting step in steroidogenesis, leading ultimately to the synthesis of estrogens [55,56]. Thus, adequate intracellular cholesterol availability is essential for maintaining estrogen levels, especially in postmenopausal women where adrenal and peripheral synthesis becomes more critical [55,56,57]. Importantly, recent evidence—including the latest meta-analysis on metabolic and inflammatory profiles in FS—has shown that elevated levels of HbA1c are a consistent feature in these patients, reflecting chronic hyperglycemia and poor glucose regulation [58]. Persistent high blood glucose can directly impair cholesterol transport mechanisms at the cellular level, particularly by glycation of apolipoproteins (e.g., ApoA1, ApoB100), dysfunction of LDL receptors, and increased oxidative modification of lipoproteins. These changes lead to decreased cholesterol uptake by cells, increased circulating LDL-C, and ultimately hypercholesterolemia [58,59,60].

This altered cholesterol metabolism not only reduces the substrate available for estrogen synthesis, potentially exacerbating hormonal imbalances, but also contributes to the formation of pro-inflammatory lipid species (e.g., oxidized LDL), which activate macrophages, endothelial cells, and fibroblasts via toll-like receptors and the NF-κB pathway [61,62]. The result is a sustained inflammatory response, fibrosis, and impaired tissue repair—a molecular landscape highly relevant to FS pathophysiology [63,64].

Furthermore, impaired glucose and cholesterol homeostasis can lead to mitochondrial dysfunction through increased production of reactive oxygen species (ROS), reduced ATP synthesis, and impaired cellular redox status [21,26,65,66]. In fibroblasts, these disruptions can activate fibrotic signaling cascades, including TGF-β1 and connective tissue growth factor (CTGF), promoting extracellular matrix (ECM) deposition and capsular thickening in the shoulder [64]. Therefore, the metabolic crosstalk between hyperglycemia, dyslipidemia, and hormonal decline may act synergistically to sustain the chronic inflammation, fibrosis, and pain characteristic of FS. Understanding and targeting this axis is crucial for developing future integrative interventions.

## 5. Estrogen Deficiency, Resistance, and Metabolic Disruption in FS

The biological relevance of estrogen in human physiology goes far beyond reproductive function [67,68]. As a pleiotropic hormone, estrogen exerts systemic effects on immune regulation, metabolism, tissue repair, vascular tone, and pain modulation [67,69,70]. These regulatory roles are especially relevant in conditions like FS, a disorder characterized by chronic capsular inflammation, fibrosis, and functional loss that disproportionately affects women, particularly during perimenopause and postmenopause (see Table 1) [5]. Despite this striking epidemiological pattern, the role of estrogen and the broader female hormonal axis has been largely overlooked in FS pathophysiology [5,15].

Emerging evidence suggests that the estrogen axis is not only vulnerable to deficiency states—as seen in menopause—but also to resistance mechanisms and metabolic interference that hinder receptor-level signaling [34,48,71]. In the context of FS, where metabolic disturbances such as chronic LGI, hyperglycemia, dyslipidemia, and adipose tissue dysfunction converge, estrogen signaling may be downregulated or altered in ways that exacerbate fibrosis, pain sensitization, and impaired tissue regeneration [21,22,24,28]. This section explores these complex interrelationships, aiming to reframe FS within a broader immunometabolic-endocrine model [14,72].

### 5.1. Estrogen Resistance and Chronobiological Disruption

While hormonal deficiency—particularly the decline in estrogen during menopause—has been widely studied, less attention has been paid to the concept of hormonal resistance, including resistance to estrogen [73]. Hormonal resistance refers to a state in which circulating hormone levels may be adequate or only mildly reduced, yet tissue responsiveness is diminished due to defects at the receptor or post-receptor level [74,75]. In the case of estrogen, this can involve decreased receptor density, impaired receptor activation, altered cofactor binding, or interference by inflammatory or metabolic signals [34]. This phenomenon may be accentuated by chronobiological dysregulation. Estrogen follows a natural circadian and infradian rhythm, with cyclical acrophases and nadirs that coordinate immune and metabolic processes [76,77,78]. Disruption of this rhythm—through aging, stress, light pollution, or metabolic dysfunction—may desynchronize estrogen receptor activation and gene transcription [77,78,79,80]. In perimenopausal women, for instance, the amplitude of estrogen fluctuations increases while predictability declines, leading to episodic surges and troughs that confuse receptor signaling machinery [81]. In FS, such dysregulation may promote an inflammatory microenvironment where estrogen cannot exert its usual anti-inflammatory, anti-fibrotic, and analgesic effects. Receptor resistance or desensitization in synovial fibroblasts, immune cells, or endothelial tissue could thus contribute to unchecked fibrosis, nociceptive amplification, and impaired tissue remodeling.

### 5.2. Hyperglycemia and AGEs

Hyperglycemia represents a major source of metabolic stress and a key factor in estrogen signaling impairment [82,83]. Chronic elevations in blood glucose—commonly assessed via HbA1c—lead to the non-enzymatic glycation of proteins, forming advanced glycation end-products (AGEs) [82,83]. AGEs are known to accumulate in connective tissue, disrupt collagen homeostasis, and activate receptors such as RAGE (Receptor for Advanced Glycation End-products), which in turn stimulate the NF-κB pathway and the release of pro-inflammatory cytokines like IL-1β and TNF-α [45,84,85].

Importantly, AGEs also interfere with lipid transport and cholesterol homeostasis, impairing the availability of cholesterol as a precursor for steroidogenesis [86]. This is particularly relevant for estrogen synthesis, which relies on sufficient intracellular cholesterol [82,86]. The sustained presence of AGEs can also oxidize LDL particles, contributing to dyslipidemia and endothelial dysfunction, both of which have been associated with FS [87,88]. Moreover, hyperglycemia-induced oxidative stress downregulates estrogen receptor expression and functionality, leading to estrogen resistance at the cellular level, even in the presence of circulating hormone. In the context of FS, this may potentiate synovial fibroblast activation and perpetuate the fibrotic cascade [89,90].

### 5.3. Dyslipidemia and Cholesterol Overload

Cholesterol plays a central role in steroid hormone biosynthesis, acting as a precursor for estrogen and other sex steroids [91]. However, in metabolic disorders like insulin resistance and FS, this balance may be disrupted [29,58,92]. Hypercholesterolemia, particularly elevated LDL and total cholesterol, has been repeatedly observed in FS patients and may act as both a marker and contributor to disease pathogenesis [58].

Cholesterol overload in tissues can lead to membrane stiffness, impaired cellular signaling, and pro-inflammatory foam cell formation [91,93]. Excess cholesterol may inhibit estrogen biosynthesis by saturating feedback loops in steroidogenic tissues or by interfering with transport proteins and enzymes such as CYP19A1 (aromatase) [53]. This creates a vicious cycle where high cholesterol contributes to low estrogen bioavailability and signaling, exacerbating inflammation and fibrosis [94].

In FS, this dyslipidemic environment may further fuel capsular thickening, neoangiogenesis, and immune cell infiltration [8,58,59]. Furthermore, oxidized LDL can interact with estrogen receptors or compete for similar binding sites, contributing to receptor-level interference and functional resistance [95].

### 5.4. Adipose Tissue Dysfunction and Endocrine Crosstalk in FS

Adipose tissue, long considered a passive energy reservoir, is now recognized as a dynamic endocrine organ that exerts profound influences on inflammation, immunity, and hormonal signaling [96]. In FS, particularly among patients with central obesity or features of metabolic syndrome, adipose dysfunction may play a central role in modulating disease course [23,97].

Hypertrophic adipocytes secrete pro-inflammatory adipokines such as leptin, resistin, and visfatin, while reducing anti-inflammatory signals like adiponectin [98,99]. These changes foster systemic LGI, which in turn impairs estrogen receptor signaling by inducing serine phosphorylation, oxidative damage, or nuclear exclusion of receptor complexes [5,100,101]. Moreover, leptin resistance—frequently seen in insulin-resistant states—has been shown to downregulate estrogen receptor alpha (ERα) in various tissues [101].

This dysfunctional adipose secretome may also affect fibroblast activity, matrix remodeling, and synovial inflammation [96,102]. Visceral adiposity, in particular, has been associated with elevated TNF-α and IL-6 levels, key drivers of fibrosis in FS [22,58]. Simultaneously, reduced aromatase activity in dysfunctional adipose tissue may decrease peripheral estrogen synthesis, compounding the effects of systemic estrogen deficiency or resistance [103,104,105].

Additionally, adipose tissue expresses estrogen receptors, and their dysfunction contributes to a feedback loop of hormonal insensitivity, metabolic imbalance, and inflammatory activation [106,107]. This establishes a complex endocrine-immuno-metabolic network in FS, where estrogen resistance becomes both a cause and consequence of adipose dysfunction [108,109].

### 5.5. Clinical and Molecular Implications in FS

The cumulative effects of estrogen resistance, chronic inflammation, hyperglycemia, dyslipidemia, and adipose tissue dysfunction converge to create a pathological environment conducive to FS development and progression [8,58,59,92]. At the molecular level, impaired estrogen signaling reduces the transcription of anti-inflammatory cytokines (e.g., IL-10), increases M1 macrophage polarization, and enhances TGF-β-mediated fibroblast activation [110]. Estrogen receptor dysfunction in joint capsules may also impair angiogenesis, increase mast cell degranulation, and limit extracellular matrix turnover [15,73]. Clinically, this translates into a more severe and prolonged disease course, particularly in women undergoing perimenopausal transition or experiencing metabolic syndrome [46,81]. These patients often present with higher pain intensity, reduced mobility, and slower response to physical therapy [92]. Recognizing these molecular signatures could facilitate early identification of high-risk phenotypes and guide tailored interventions—for instance, combining hormonal modulation with anti-inflammatory diets, exercise, and insulin-sensitizing agents.

As the field progresses, integrating estrogen signaling pathways into FS management could offer a more comprehensive framework for understanding and treating this complex condition.

### 5.6. Estrogen Deficiency in Menopause: A Missing Link in FS

Estrogen deficiency has traditionally been considered one of the principal hormonal drivers behind the disproportionately high prevalence of FS in women, particularly during the perimenopausal and postmenopausal stages [46]. During this transitional period, ovarian estrogen production declines significantly, often to below 10% of premenopausal levels [15]. Far from being a solely reproductive hormone, estrogen plays a vital role in maintaining immune homeostasis, connective tissue remodeling, metabolic regulation, and nociceptive modulation [48,70,108]. Its absence introduces a cascade of dysregulated processes that can critically influence the onset and progression of FS [58].

At the molecular level, 17β-estradiol (E2), the most potent form of estrogen, performs several protective functions highly relevant to FS pathogenesis:

Anti-inflammatory effects: Estrogens inhibit key pro-inflammatory transcription factors, notably nuclear factor kappa B (NF-κB), thereby reducing the expression of cytokines such as IL-1β, IL-6, and TNF-α. These mediators are commonly elevated in synovial fluid and capsular biopsies from FS patients and are involved in promoting nociceptive sensitization and fibrotic responses [20,27,34,111].

Antifibrotic signaling: E2 modulates the transforming growth factor-beta (TGF-β1) pathway, a central orchestrator of fibrotic remodeling. In physiological conditions, estrogen limits fibroblast-to-myofibroblast transition and curbs collagen I/III synthesis. Its deficiency allows unopposed TGF-β1 activity, resulting in capsular thickening, contracture, and loss of joint range [31,48,49,69].

Antioxidant defense: Estrogen promotes the expression of antioxidant enzymes such as superoxide dismutase (SOD), catalase, and glutathione peroxidase, thereby reducing oxidative stress. The accumulation of reactive oxygen species (ROS) in low-estrogen environments exacerbates collagen cross-linking, impairs angiogenesis, and accelerates extracellular matrix stiffening [112].

Pain regulation: Estrogens modulate both central and peripheral pain pathways through their interaction with opioid receptors, TRPV1 channels, and NMDA receptors. Estrogen deficiency may lead to heightened pain sensitivity and decreased endogenous analgesia—hallmarks of FS symptomatology [41,113].

Histological studies have confirmed the presence of estrogen receptors (ERα and ERβ) in the glenohumeral joint capsule, particularly in fibroblasts, endothelial cells, and synovial lining. When estrogen is deficient, these receptors may remain unbound and inactive, disrupting normal gene transcription for anti-inflammatory and tissue-reparative processes [114].

The downstream consequences of this signaling void include: (i) Enhanced fibroblast proliferation and extracellular matrix deposition [31,115]; (ii) Sustained activation of pro-inflammatory M1 macrophages and reduced IL-10 production [116,117]; (iii) Altered expression of matrix metalloproteinases (MMPs) and their inhibitors (TIMPs), impairing collagen turnover [26,66]; (iv) Increased vascular permeability and neoangiogenesis, promoting synovial hyperplasia [118].

Clinically, estrogen deficiency is associated with greater FS severity in postmenopausal women, who often experience more intense pain, prolonged stiffness, and delayed response to rehabilitation. The correlation between low estrogen and other musculoskeletal disorders—such as osteoarthritis, tendinopathy, and sarcopenia—further reinforces its role as a central modulator of connective tissue health and repair [15]. Notably, estrogen deficiency seldom occurs in isolation. It often coexists with increased insulin resistance, visceral adiposity, and elevated LDL cholesterol, all of which converge to reinforce the fibrotic and inflammatory milieu [58]. Moreover, the decline in aromatase activity within dysfunctional adipose tissue reduces peripheral estrogen production, amplifying systemic deficiency [104]. However, it is also important to recognize that not all postmenopausal women develop FS, and cases in premenopausal women suggest that deficiency alone is insufficient to trigger the condition [15]. This observation has led to growing interest in the roles of estrogen resistance, receptor-level interference, and metabolic stress as complementary or alternative mechanisms—paving the way for more nuanced and integrative models of FS pathogenesis [5,8,58,59].

### 5.7. Receptor-Level Interference by Metabolic and Environmental Factors

In recent years, a growing body of evidence has emphasized that endocrine signaling—particularly estrogen signaling—can be profoundly disrupted not only by internal pathophysiological changes, but also by chronic exposure to environmental, dietary, and lifestyle factors [119,120]. This subclinical disruption can occur even in the presence of normal or mildly reduced estrogen levels, and is increasingly understood as a central mechanism in the pathogenesis of hormone-sensitive disorders such as FS [71,73].

#### 5.7.1. Environmental Estrogen Disruptors and Receptor Occupancy

Modern industrial environments expose individuals to a wide range of endocrine-disrupting chemicals (EDCs) [121,122]. These include xenoestrogens, such as bisphenol A (BPA), phthalates, parabens, polychlorinated biphenyls (PCBs), and heavy metals (e.g., cadmium), which are ubiquitously found in plastics, food packaging, cosmetics, detergents, pesticides, and even indoor air [122]. These compounds mimic the structure of natural estrogens and bind to estrogen receptors (ERα and ERβ), acting as partial agonists or antagonists depending on the tissue [121]. Although some EDCs weakly activate estrogen receptors, their constant low-level presence leads to receptor desensitization, misactivation, or competitive inhibition, which interferes with the body’s endogenous estrogen signaling [123]. In fibroblasts, immune cells, or endothelial cells within the glenohumeral capsule, this interference could blunt the normal anti-inflammatory and antifibrotic actions of estrogen, promoting unchecked cytokine release, immune cell recruitment, and matrix stiffening—hallmarks of FS pathology [124,125].

#### 5.7.2. Chronic Low-Grade Inflammation (LGI) and Receptor Dysfunction

A pro-inflammatory lifestyle, characterized by poor diet, physical inactivity, chronic psychological stress, inadequate sleep, and environmental pollutant exposure, can induce a sustained state of LGI [126,127]. Unlike acute inflammation, LGI operates at subclinical levels, often undetected by routine diagnostics, but it exerts pervasive molecular effects [128]. LGI activates transcription factors such as NF-κB and AP-1, which not only promote cytokine and chemokine expression (e.g., IL-6, TNF-α, IL-1β), but also impair estrogen receptor signaling through multiple mechanisms [129]: (i) Serine phosphorylation of ERα, which reduces its transcriptional activity and may even convert it into a pro-inflammatory modulator; (ii) Nitrosative and oxidative stress, which modifies receptor structure, reducing ligand binding affinity and nuclear translocation; (iii) Downregulation of receptor expression, particularly in adipose, immune, and musculoskeletal tissues; (iv) This inflammatory-induced ER dysfunction renders peripheral tissues partially or fully resistant to estrogen, even in the presence of normal hormone concentrations. In FS, this would hinder estrogen’s regulation of fibroblast activation, immune polarization, and vascular function, thereby fueling capsular fibrosis and pain sensitivity [72,125,126,130,131].

#### 5.7.3. Metabolic Disruptors: Diet, Obesity, and Mitochondrial Stress

Modern dietary patterns—rich in ultra-processed foods, refined carbohydrates, trans fats, and artificial additives—are a major driver of metabolic dysregulation [132,133]. Diets with high glycemic load, poor fiber diversity, and excessive omega-6 to omega-3 ratios promote insulin resistance, adipose tissue dysfunction, and microbiota imbalances, all of which converge on inflammatory and endocrine axes [134,135,136]. These metabolic shifts result in: (i)Elevated leptin and decreased adiponectin, which impair ERα expression [137]; (ii) Mitochondrial dysfunction and ROS accumulation, damaging estrogen receptors and coactivators [138,139]; (iii) Reduced cholesterol bioavailability for steroidogenesis, particularly under conditions of dyslipidemia and AGE accumulation [61,140,141]. Moreover, high-fat, high-sugar diets exacerbate hepatic estrogen metabolism, leading to increased conjugation and excretion, thereby reducing systemic bioavailability of active estrogens [48,142,143]. The result is a state of functional estrogen deficiency, even without absolute hormonal decline [71].

#### 5.7.4. Hormonal Crosstalk and Receptor Interference

Insulin, cortisol, leptin, and inflammatory cytokines all interact with estrogen signaling pathways, either by competing for shared coactivators (e.g., SRC-1, p300) or by modulating transcriptional responses at estrogen-responsive elements (EREs) [144]. Chronic insulin resistance and hypercortisolemia, common in FS patients with metabolic syndrome, may further inhibit estrogen action through: (i) Nuclear receptor crosstalk, where glucocorticoid receptor activation suppresses ER-mediated transcription [145]; (ii) Histone deacetylase activation, which closes chromatin at ER target sites [146]; (iii) Direct inhibition of aromatase, reducing local estrogen synthesis in tissues such as fat and muscle [147]. This complex hormonal crosstalk means that receptor functionality is not only a function of estrogen concentration, but also of the metabolic and inflammatory milieu, which can override or block hormonal effects [71].

## 6. A Paradigm Shift in FS Pathophysiology

The traditional understanding of FS has long centered on localized mechanical and inflammatory changes within the glenohumeral joint capsule—namely, synovitis, fibroblast proliferation, and collagen deposition [1,2,22]. While this model has clinical utility, it fails to account for the striking epidemiological, metabolic, and hormonal patterns increasingly observed in FS patients, particularly in women during the menopausal transition [46,58]. A growing body of evidence now calls for a paradigm shift: one that moves beyond joint-centric explanations and instead views FS as a systemic, immunoendocrine-metabolic disorder [5,8,14,25,50,58,124]. In this revised framework, the shoulder capsule becomes not the origin, but the target organ of upstream dysregulations involving sex hormones, chronic LGI, metabolic dysfunction, and environmental interference [5,8,14,25,50,58,124].

Estrogen, once considered solely a reproductive hormone, now emerges as a central regulatory signal for immune modulation, extracellular matrix remodeling, pain processing, and tissue repair [148,149]. Its deficiency, resistance, or receptor-level disruption—whether due to menopause, metabolic syndrome, or environmental exposures—sets the stage for a pathological cascade where tissue resilience is lost and [150,151] fibrosis prevails. At the same time, systemic factors such as hyperglycemia, dyslipidemia, mitochondrial stress, and adipokine imbalance exacerbate this hormonal vulnerability [152]. They promote fibro-inflammatory signaling through the activation of TGF-β, NF-κB, and pro-inflammatory macrophages, while simultaneously blunting protective pathways involving IL-10, antioxidant enzymes, and estrogen receptor signaling [153]. The result is a pro-fibrotic, pro-inflammatory phenotype, particularly evident in women with features of metabolic stress or hormonal transition [72,124]. Furthermore, the occupational burden of endocrine-disrupting chemicals (EDCs) and environmental stressors (e.g., sleep deprivation, circadian disruption, psychosocial stress) adds a previously overlooked dimension to FS pathogenesis [154,155]. These factors interfere with receptor binding, chronobiological hormone release, and intracellular signaling fidelity, making estrogen resistance a likely—and perhaps central—player in disease expression [121].

**Figure 1:**
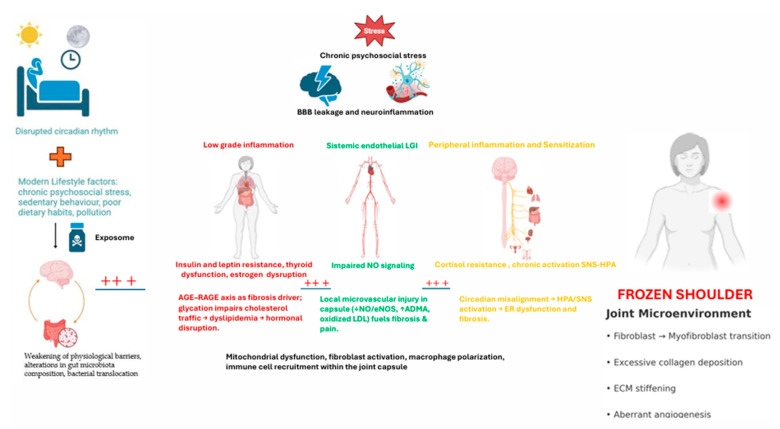
Integrative model of frozen shoulder pathophysiology. This figure illustrates the multifactorial cascade linking lifestyle, environmental, and systemic factors to the onset and persistence of FS. On the left, modern lifestyle influences—including circadian disruption, sedentarism, poor dietary habits, pollution, and broader exposome exposures—act as primary triggers of systemic dysregulation. These exposures weaken physiological barriers, alter gut microbiota composition, and promote bacterial translocation, thereby amplifying inflammatory signaling. In the central section, chronic psychosocial stress contributes to blood–brain barrier leakage and neuroinflammation, which interact with systemic pathways represented in three axes: (i) low-grade systemic inflammation (LGI), encompassing endocrine and metabolic alterations such as insulin and leptin resistance, thyroid dysfunction, and estrogen dysregulation (red text); (ii) systemic endothelial inflammation, marked by vascular injury, impaired nitric oxide (NO) signaling, and microvascular dysfunction (green text); and (iii) peripheral inflammation and sensitization, characterized by chronic activation of the sympathetic nervous system, hypothalamic–pituitary–adrenal axis, and cortisol resistance (yellow text). Collectively, these processes converge downstream to promote mitochondrial dysfunction, fibroblast activation, macrophage polarization, and immune cell recruitment within the joint capsule. The final right-hand representation highlights the clinical phenotype of FS, where fibroblast-to-myofibroblast transition, excessive collagen deposition, extracellular matrix stiffening, and aberrant angiogenesis result in capsular fibrosis, chronic pain, and restricted mobility.

This integrative lens compels us to re-evaluate not only the causes of FS but also the way it is treated. If FS were, in part, a manifestation of disrupted estrogen signaling within a pro-inflammatory metabolic environment, then future therapeutic approaches must go beyond joint mobilization or corticosteroids. They should include: (i) Hormonal evaluation and, where appropriate, modulation (e.g., estrogen support, phytoestrogens); (ii) Anti-inflammatory nutritional interventions; (iii) Lifestyle optimization focused on circadian alignment, stress management, and physical activity; (iv) Environmental detoxification strategies to reduce EDC exposure; (v) Targeted metabolic interventions to restore insulin sensitivity, mitochondrial health, and lipid balance. Thus, the future of FS management lies not only in better understanding the joint capsule—but in restoring systemic balance at the intersection of hormones, metabolism, immunity, and environment. Such a paradigm shift holds promise for not only improved treatment outcomes but also for preventive strategies, particularly in high-risk female populations. To facilitate comprehension and provide a visual synthesis of the integrative model proposed, a summary figure (Figure 1) was developed. This schematic representation illustrates how lifestyle, endocrine, metabolic, vascular, and neuroimmune factors interact to create a systemic environment that culminates in capsular fibrosis, pain sensitization, and the clinical phenotype of frozen shoulder.

### 6.1. Sleep, Circadian Rhythms, and Hormonal Crosstalk in FS

The endocrine system operates not in isolation but in harmony with the body’s circadian architecture, governed by the suprachiasmatic nucleus (SCN) of the hypothalamus. This master clock orchestrates daily rhythms of hormonal release—including estrogen, melatonin, cortisol, thyroid hormones, and growth hormone (GH)—which in turn regulate immune tone, metabolic efficiency, tissue repair, and inflammatory resolution [156,157]. Disruption of these rhythms, particularly through poor sleep quality or altered light exposure, has profound implications for musculoskeletal health and may be a silent driver of FS pathogenesis [29,158].

Sleep is essential for hormonal synchronization, with specific peaks in anabolic and regulatory hormones occurring during nocturnal rest [159,160]. For instance: (i) Estrogen secretion, although modulated primarily by the ovarian axis, follows ultradian rhythms influenced by circadian cues. Fragmented or delayed sleep impairs estrogen receptor activation and downstream gene transcription [159]; (ii) Growth hormone, critical for collagen turnover and connective tissue repair, is secreted in pulses during slow-wave sleep. Poor sleep reduces GH amplitude, impairing tendon and capsular recovery [161]; (iii) Cortisol, with its diurnal peak in the early morning, may become chronically elevated with sleep disruption, contributing to insulin resistance, immunosuppression, and estrogen resistance [162,163]; (iv) Melatonin, the principal chronobiotic hormone, modulates estrogen receptor expression and has anti-fibrotic, antioxidant, and immune-regulating properties [164,165]. Light-at-night, especially from screens, suppresses melatonin production and indirectly derails hormonal coordination [166]; (v) Disrupted sleep architecture thus acts as a multisystemic stressor, capable of precipitating or worsening LGI, estrogen insensitivity, and metabolic imbalance—all of which converge in the FS phenotype [5,14,167].

### 6.2. Thyroid Dysfunction in FS: A Key but Overlooked Axis

Among the endocrine alterations associated with FS, thyroid dysfunction has received increasing attention [168]. Population-based and clinical studies have reported a higher prevalence of hypothyroidism in FS patients, with estimates ranging from 10% to 34% [168,169]. A recent systematic review and meta-analysis confirmed that both overt and subclinical hypothyroidism significantly increase the risk of FS, whereas the evidence for hyperthyroidism is less consistent [170]. Indeed, a Mendelian randomization study has provided causal support for the association between hypothyroidism and FS, suggesting a direct role of thyroid hormones in capsular pathophysiology [9].

Several mechanisms have been proposed. Hypothyroidism reduces basal metabolic rate and mitochondrial function, promotes the accumulation of glycosaminoglycans, and disrupts collagen turnover, thereby favoring capsular fibrosis [171]. In addition, reduced expression of matrix metalloproteinases (MMPs) and increased TGF-β have been reported, stimulating fibroblast-to-myofibroblast transition and extracellular matrix accumulation [171]. In parallel, thyroid hormone deficiency may induce insulin resistance and alterations in leptin and adiponectin, interfering with estrogen signaling and contributing to a systemic pro-inflammatory state. In patients with autoimmune thyroiditis (e.g., Hashimoto’s disease), the presence of chronic inflammatory background may further amplify local fibrotic processes in the joint capsule [172,173]. Although hypothyroidism is the condition most frequently associated with FS, some recent studies suggest that a hypermetabolic state may also be implicated [8]. In this context, increased oxidative stress and metabolic demand could potentiate inflammatory responses and pain sensitization [8,59]. These findings indicate that both deficient and excessive thyroid hormone states may alter the capsular microenvironment, albeit through different mechanisms.

Clinically, the coexistence of FS and thyroid dysfunction is often associated with greater symptom severity and, in some cases, with poorer response to conventional treatment [58,170]. However, results remain inconsistent, and further studies are required to determine whether correcting thyroid dysfunction directly improves FS outcomes [168,170]. Importantly, both thyroid hormones (T3 and T4) and estrogens act in coordination to regulate metabolism, connective tissue remodeling, immune modulation, and pain perception [174,175]. When one of these axes is altered, the other is often secondarily affected, creating an environment favorable to chronic inflammation and fibrosis. In this context, the estrogen axis is deeply impacted [174,175]. Hypothyroidism alters the synthesis of sex hormone–binding globulin (SHBG), reducing estrogen bioavailability, while also inducing insulin and leptin resistance, which interfere with estrogen receptor signaling [174,176]. This combination can lead to functional estrogen resistance, even in the presence of relatively preserved serum estrogen levels [176]. Since estrogens exert anti-inflammatory, antioxidant, and antifibrotic properties—such as inhibiting NF-κB and negatively regulating TGF-β—their dysfunction leaves the joint microenvironment more vulnerable to damage [34,42,49,151,177].

The interaction between estrogen deficiency (due to menopause, resistance, or signaling alterations) and thyroid dysfunction creates a vicious cycle that exacerbates pain and fibrosis: (i) Hypothyroidism promotes fibrosis and pain sensitization through excessive TGF-β activity, impaired mitochondrial function, and systemic inflammation [178]; (ii) Estrogen deficiency or resistance prevents counteracting these mechanisms by limiting fibroblast regulation, reducing antioxidant activity (SOD, catalase, GPx), and diminishing central pain modulation [179]; (iii) Together, these dysfunctions converge into an inflammatory-fibrotic phenotype, characterized by M1 macrophage activation, excess ROS, and disrupted collagen metabolism [180,181].

Clinically, this scenario is particularly evident in peri- and postmenopausal women, where the prevalence of subclinical or overt hypothyroidism is higher and the decline in estrogen levels is more pronounced [9,15]. In this group, FS tends to present with more intense pain, greater capsular stiffness, and poorer response to conventional treatments [10,170]. Thus, the combined dysfunction of the thyroid and estrogen axes may constitute a central node in the pathogenesis of FS. Their disruption not only promotes the establishment of fibrosis and chronic pain, but also provides a plausible explanation for the strong female predominance of FS and its frequent association with metabolic comorbidities.

### 6.3. The Lifestyle Hypothesis: LGI as the Root Cause

An increasing body of evidence suggests that many endocrine and metabolic derangements in FS may originate from lifestyle-induced chronic LGI [127,132,182]. Modern behaviors—sedentarism, diets rich in ultra-processed foods and poor in micronutrients, excessive screen exposure and artificial light-at-night (ALAN), psychosocial stress, and sleep disruption—collectively impair circadian biology, induce persistent immune activation, and promote hormonal resistance across multiple axes [132].

LGI operates through sustained activation of stress axes (HPA, HPT, HPG) and inflammatory mediators [72,127]. This systemic inflammatory context leads to: (i) Insulin resistance, hyperglycemia, and AGEs, which desensitize estrogen receptors and enhance fibrotic signaling [48,183]; (ii) Leptin resistance, impairing hypothalamic–pituitary regulation and disrupting estrogen and GH rhythms [184,185]; (iii) Cortisol dysregulation, with chronic elevation suppressing melatonin and TSH, thereby compromising both thyroid and estrogen pathways [162,186,187]; (iv) Oxidative and nitrosative stress, driving receptor phosphorylation, conformational changes, and nuclear exclusion of estrogen–receptor complexes [188].

Psychoneuroimmunology research highlights how these contextual risk factors act as “lifestyle-associated molecular patterns” (LAMPs) that mimic pathogen or danger signals, thereby perpetuating immune activation even in the absence of acute threats [126]. This maladaptive response—robust in evolutionary terms but deleterious in modern environments—may explain why FS manifests as a systemic immunoendocrine disorder rather than a purely local musculoskeletal disease [5,8,14,58]. Thus, the FS phenotype could be reframed as the clinical expression of endocrine–metabolic disintegration rooted in lifestyle and environmental exposures. Recognizing this cascade underscores the urgency of adopting integrative interventions, such as sleep hygiene, stress resilience training, anti-inflammatory and phytonutrient-rich nutrition, regular physical activity, and targeted hormonal rebalancing. These strategies should not be considered ancillary, but rather core therapeutic pillars in the management and prevention of FS.

On the other hand, socio-emotional health and sustained psychosocial stress appear to play a significant role in both the onset and perpetuation of FS symptoms [8]. Recent systematic reviews have shown that pain-related fear and depression are prognostic for disability, function, and pain severity, while anxiety is consistently associated with worse self-reported pain and functional impairment [3]. In perioperative settings, prospective data indicate that preoperative anxiety increases the risk of developing FS after rotator cuff repair, underscoring the importance of psychological factors in secondary shoulder stiffness [30]. From a psychoneuroimmunological perspective, chronic stress sustains HPA axis activation and sympathetic nervous system drive, fostering neuroinflammation, LGI, and increased barrier permeability (e.g., intestinal), all of which amplify systemic immune activation [14]. In FS specifically, psychosocial stressors, circadian disruption, and lifestyle-related factors have been proposed as converging triggers of LGI, fibrotic signaling, and pain sensitization [5,8,14,25,58]. Practically, integrating psychological assessment and targeted interventions—addressing anxiety, depression, catastrophizing, and fear of movement—into rehabilitation may enhance treatment outcomes [3]. This is supported by evidence on the benefits of psychological strategies and pain neuroscience education in musculoskeletal pain and shoulder disorders [189,190,191].

#### The Lifestyle Hypothesis: Endothelial Health

Finally, another key pathway linking lifestyle factors to FS pathogenesis is endothelial inflammation and dysfunction [3,30,192,193]. Modern exposures such as poor nutrition, ultra-processed diets, sedentarism, chronic psychosocial stress, sleep disruption, and environmental pollutants elevate circulating inflammatory mediators, homocysteine, oxidized lipids, and particulate toxins [132,194,195]. These factors directly injure the vascular endothelium, leading to reduced nitric oxide (NO) bioavailability and impaired endothelial nitric oxide synthase (eNOS) activity [196,197]. The imbalance between vasodilatory and vasoconstrictive forces promotes oxidative and nitrosative stress, driving the generation of peroxynitrite and other reactive nitrogen species [198,199]. This state of endothelial dysfunction sustains systemic low-grade inflammation while simultaneously producing local microvascular alterations in the shoulder capsule, including impaired perfusion, increased vascular permeability, leukocyte recruitment, and aberrant angiogenesis [26,118]. These events create a permissive environment for fibroblast activation, excessive collagen deposition, and ultimately capsular fibrosis and persistent pain (see Table 2) [22,26].

In parallel, lifestyle-driven disturbances in the gut microbiota further amplify vascular and immune dysregulation [200,201,202]. Dysbiosis and increased intestinal permeability promote bacterial translocation and the systemic release of pro-inflammatory metabolites, such as lipopolysaccharides (LPS) and asymmetric dimethylarginine (ADMA), the latter being a potent endogenous inhibitor of eNOS [203,204]. Elevated ADMA levels have been strongly associated with impaired NO production, vascular inflammation, and collagen remodeling [204]. In FS, this may synergize with capsular hypoperfusion and aberrant angiogenesis, fostering extracellular matrix stiffening and reduced tissue resilience [118].

The role of AGEs adds another mechanistic layer to this process [82,83]. Chronic hyperglycemia and oxidative stress drive AGE accumulation in connective tissue, where they cross-link collagen fibers, reduce tissue elasticity, and impair remodeling [205]. AGEs also bind to their receptor (RAGE) on endothelial and immune cells, activating NF-κB signaling and perpetuating a cycle of cytokine release, vascular inflammation, and fibroblast proliferation [84,206]. Recent studies in idiopathic FS have confirmed elevated AGE deposition in the shoulder capsule, supporting their pathogenic contribution not only as markers of chronic metabolic imbalance but as active mediators of fibrosis [21,24].

Altogether, these interrelated mechanisms illustrate how lifestyle-related exposures converge at the vascular interface to initiate a primary inflammatory insult at the endothelium [199,207]. This “first hit” propagates into systemic LGI, disrupts hormonal and metabolic regulation, and establishes a molecular milieu favoring fibrosis, pain sensitization, and chronic joint dysfunction [72,127]. Within FS, the endothelial–immune axis therefore represents a critical but underrecognized bridge between lifestyle behaviors, metabolic stress, and the characteristic clinical phenotype of stiffness and pain (see Table 3) [14,58,83].

## 7. Future Research Directions

The integration of hormonal, metabolic, vascular, and lifestyle factors into the pathophysiological framework of FS underscores the need for a paradigm shift in research. Historically, FS has been examined through orthopedic and localized inflammatory lenses; however, converging evidence supports FS as a systemic immuno–endocrine–metabolic condition—particularly in women—best studied with systems-biology approaches. To provide practical guidance to the field, we delineate short-term, feasible directions and long-term, exploratory programs.

### 7.1. Short-Term, Feasible Directions (12–24 Months)

**(A)**  **Longitudinal endocrine monitoring in routine care**

Dynamic profiling of estradiol, progesterone, testosterone, cortisol, thyroid hormones (TSH, T3, T4), leptin, insulin, and melatonin across menopausal and metabolic states to reveal patterns that precede or accompany capsular fibrosis and pain chronification. Couple these measures to pain, function, ROM, and time-to-recovery to generate actionable phenotyping rules.

**(B)**  **Endothelial–vascular assessments in FS**

Evaluate microvascular flow surrogates together with nitric-oxide metabolism (e.g., NO metabolites, asymmetric dimethylarginine [ADMA]) and endothelial inflammation (e.g., ICAM-1/VCAM-1). Clarify how lifestyle, dysbiosis, and metabolic stress converge on the endothelium to trigger capsular fibrosis.

**(C)**  **Sleep and circadian alignment pilots**

Use actigraphy plus melatonin/cortisol rhythm analysis to quantify circadian misalignment and test pragmatic interventions (sleep hygiene, evening blue-light restriction, timed light therapy, melatonin where appropriate) on pain, function, and ROM.

**(D)**  **Diet-first pragmatic trials**

Pilot integrative nutritional strategies—Mediterranean, anti-inflammatory, low-AGE, phytoestrogen-supportive or ketogenic diets—embedded in rehabilitation. Track adherence, HbA1c, lipid fractions, inflammatory mediators, and patient-reported outcomes.

**(E)**  **Focused micronutrient add-on trials (women-centric)**

Test vitamin D, manganese, and selected B-vitamins (e.g., B1/B3) where observational signals suggest benefit, using short randomized add-on designs to determine effect sizes and feasibility.

**(F)**  **Integrative biomarker panels for stratification**

Assemble compact panels combining inflammatory (IL-1β, IL-6, TNF-α, HMGB1), metabolic (HbA1c, HOMA-IR, lipid fractions, AGEs), hormonal (estradiol, SHBG, thyroid function) and vascular markers (NO metabolites, ADMA, ICAM-1/VCAM-1) to classify FS subtypes by systemic dysfunction rather than symptoms alone.

### 7.2. Long-Term, Exploratory Programs (24–60+ Months)

**(G)**  **Estrogen deficiency/resistance and receptor-level interference**

Undertake tissue and liquid-biopsy studies to assess estrogen receptor density, nuclear localization, phosphorylation status, and downstream signaling (ERK, PI3K/Akt). Determine whether receptor dysfunction causally links to fibroblast activation, immune dysregulation, and impaired matrix repair.

**(H)**  **Systems mapping of the vascular–immune–fibrotic axis**

Combine vascular imaging/surrogates, NO/ADMA biology, cytokine profiling, and shoulder imaging (US/MRI) to model causal pathways from endothelial dysfunction to fibrosis and pain sensitization.

**(I)**  **Chronobiology and sleep as disease modifiers**

Run mechanistic trials aligning sleep/wake timing and light exposure with endocrine outputs (estrogen, GH, thyroid, cortisol) to test whether circadian restoration modifies immuno-metabolic signatures and clinical trajectories.

**(J)**  **Gut microbiota and the gut–vascular–joint axis**

Integrate microbiome sequencing and intestinal permeability assays with metabolomics (including LPS-related and methylarginine pathways) to delineate diet–microbiome–endothelium crosstalk that impairs estrogen and NO signaling.

**(K)**  **Multi-omics and precision medicine frameworks**

Deploy metabolomic, transcriptomic, and epigenetic profiling to define FS endotypes, enabling adaptive trials that match interventions (dietary, circadian, endothelial support, hormone modulation when appropriate) to molecular phenotypes.

**(L)**  **Scalable interventional platforms and implementation science**

Progress from efficacy to effectiveness via pragmatic, multi-arm platforms in real-world PT/orthopedic settings; include cost-effectiveness, equity, and adoption metrics to support health-system uptake.

Therefore, FS should no longer be approached as an isolated orthopedic disease but as a systemic disorder in which vascular dysfunction, hormonal imbalance, metabolic stress, and lifestyle exposures converge. A staged research agenda—pairing near-term, pragmatic studies with ambitious, systems-level programs—can deliver stratified, precision-based therapies that target the underlying immuno–endocrine–vascular networks rather than the symptomatic shoulder alone.

To provide a more visual and practice-oriented synthesis of the evidence reviewed, Table 4 summarizes the key clinical signals, underlying mechanistic drivers, and proposed interventions in FS. By distinguishing between established findings supported by clinical studies and emerging hypotheses drawn from experimental or mechanistic research, this table offers a concise framework that bridges basic science and clinical management. Its purpose is to guide clinicians and researchers toward integrative strategies that combine endocrine, metabolic, vascular, and lifestyle perspectives, facilitating translation of complex concepts into practical applications.

## 8. Clinical Implications

The emerging model of FS as a systemic, hormonally modulated inflammatory–fibrotic condition reframes this disorder far beyond a localized joint pathology. In particular, receptor-level dysfunction of estrogen—exacerbated by menopause, metabolic syndrome, thyroid imbalance, endothelial dysfunction, or chronic environmental burden—may explain the greater pain severity, poor responsiveness to rehabilitation, and increased recurrence observed in many patients.

Accordingly, treatment strategies should not remain confined to orthopedic interventions but integrate metabolic, endocrine, vascular, and lifestyle dimensions. Key implications include:

1. Hormonal, metabolic, and vascular evaluation. Routine assessment of sex hormones (estradiol, progesterone, testosterone), thyroid function, cortisol rhythms, HbA1c, lipid profile, inflammatory mediators (e.g., IL-6, TNF-α), and vascular health (NO metabolites, ADMA, ICAM-1/VCAM-1) should be considered, particularly in women over 40 or in patients with metabolic risk factors.
2. Lifestyle assessment and modification. Screening for sleep quality, circadian rhythm disruption, stress levels, physical activity, and dietary habits must become part of FS evaluation. Personalized interventions may include stress reduction strategies (e.g., mindfulness, CBT), structured physical activity programs, and dietary approaches such as anti-inflammatory, low-AGE, or phytoestrogen-rich diets to support estrogen signaling, endothelial health, and systemic metabolic balance.
3. Targeting environmental and metabolic disruptors. Reducing exposure to endocrine-disrupting chemicals (e.g., BPA, phthalates, heavy metals) and addressing insulin and leptin resistance are essential for restoring endocrine sensitivity. Equally important is supporting mitochondrial function and reducing oxidative stress through antioxidant therapies, which may help normalize estrogen receptor activity and endothelial nitric oxide signaling.
4. Gut–vascular–immune axis modulation. Given growing evidence of the role of dysbiosis and intestinal permeability in systemic inflammation, therapeutic approaches including prebiotics, probiotics, and microbiome-targeted nutrition could be explored as adjunctive strategies to mitigate endothelial dysfunction, reduce ADMA levels, and restore immune tolerance.
5. Interdisciplinary management. Optimal care requires collaboration between orthopedic specialists, endocrinologists, physiotherapists, and nutritionists. Selected patients may benefit from hormonal support (e.g., bioidentical hormone therapy), correction of subclinical hypothyroidism, or integrative metabolic therapies following individualized risk–benefit evaluation.
6. Patient education. Empowering patients with knowledge about how nutrition, sleep, stress, hormonal balance, and environmental exposures influence their condition can foster adherence to lifestyle changes, strengthen self-management, and improve long-term outcomes.

Ultimately, this broader perspective reframes FS as an endocrine–metabolic–vascular musculoskeletal disorder, highlighting the need for integrative and personalized interventions that combine rehabilitation with hormonal, nutritional, vascular, and lifestyle strategies—going far beyond intra-articular corticosteroids or manual therapy alone. In order to enhance clinical applicability, Table 5 presents a structured overview of recommended assessments and potential interventions for FS, categorized by mechanistic domain and graded according to available evidence or expert consensus. This table is designed to serve as a practical tool for clinicians, highlighting how evaluation of hormonal, metabolic, vascular, and lifestyle factors can be integrated into standard FS management. By aligning clinical assessment with targeted interventions, it emphasizes the need for an interdisciplinary and personalized approach that extends beyond conventional orthopedic care.

## 9. Limitations

Because this is a narrative–scoping review, we did not track database-wise record counts or include a PRISMA flow diagram. While this limits replicability at the level of numeric yields, it is consistent with the goals and conventions of narrative synthesis. We mitigated this by (i) pre-specifying mechanistic domains and eligibility logic, (ii) documenting sources and date limits, and (iii) separating clinical and experimental evidence in text and tables. A future systematic review could extend our framework with comprehensive retrieval and quantitative flow reporting.

## 10. Conclusions

Emerging evidence suggests that FS may extend beyond a purely local capsular disorder toward a systemic, immunometabolic–vascular condition. Across the literature reviewed, dysfunction of the estrogen axis—via deficiency, resistance, or receptor-level interference—appears to act as a key amplifier of fibrosis, nociception, and impaired tissue repair, particularly in women. This hormonal vulnerability often coexists with hyperglycemia and AGEs, dyslipidemia, adipose and thyroid dysfunction, endothelial injury, gut dysbiosis, circadian and sleep disruption, psychosocial stress, and broader exposome burdens. Together, these factors sustain low-grade inflammation and fibro-inflammatory signaling that characterize the FS phenotype of pain and stiffness. Clinically, this points to the need to look beyond strictly orthopedic approaches and incorporate metabolic and hormonal profiling (estradiol, thyroid function, cortisol rhythms, HbA1c, lipid fractions, inflammatory markers) together with endothelial health surrogates, alongside rehabilitation. Integrative strategies such as anti-inflammatory and estrogen-supportive nutrition, sleep and circadian hygiene, stress-reduction approaches, and risk-adapted endocrine support in selected cases, combined with patient education, may improve outcomes and adherence. Future research should prioritize longitudinal endocrine monitoring linked to pain and function, compact diet-first and circadian/sleep interventions embedded in rehabilitation, and pilot studies of endothelial and microvascular markers. Longer-range work should address estrogen-resistance mechanisms in joint-relevant tissues, map the vascular–immune–fibrotic axis with multi-omic tools, and develop precision endotypes that enable phenotype-matched interventions. By reframing FS within a broader neuro–endocrine–immune–metabolic–vascular model, new opportunities may arise for more effective, personalized, and preventive care—particularly for peri- and postmenopausal women who remain disproportionately affected.

## Figures and Tables

**Table 1:** Estrogen & Thyroid Axes in Frozen Shoulder.

Domain	Study Type	Core Finding	Implication for FS
Estrogen axis	Clinical (SR/MA, cohorts)	Higher prevalence and worse outcomes in peri/postmenopausal women; metabolic comorbidity associations; ER expression in glenohumeral capsule biopsies.	Estrogen deficiency/resistance may underlie inflammation, pain, and fibrosis.
Estrogen axis	Experimental (in vitro/animal)	E2 inhibits NF-κB, IL-1β, IL-6, TNF-α; modulates TGF-β1 and MMP/TIMP; antioxidant and antinociceptive effects.	Supports hypothesis of receptor-level resistance in FS.
Thyroid axis	Clinical (SR/MA, MR, cohorts)	Overt and subclinical hypothyroidism ↑ risk of FS; causal support from Mendelian randomization; weaker evidence for hyperthyroidism.	Thyroid dysfunction contributes to fibrosis and pain; screening recommended.
Thyroid axis	Experimental (animal/in vitro)	Low T3/T4 → ↓MMPs, ↑TGF-β, ECM accumulation, impaired collagen turnover.	Mechanistic link between hypothyroidism and capsular fibrosis.
Estrogen–Thyroid crosstalk	Translational	Hypothyroidism alters SHBG, insulin and leptin resistance → reduced estrogen bioavailability/action.	Functional estrogen resistance in thyroid dysfunction.

Note: FS = Frozen Shoulder; SR = Systematic Review; MA = Meta-analysis; MR = Mendelian Randomization; ER = Estrogen Receptor; E2 = 17β-estradiol; NF-κB = Nuclear Factor kappa-light-chain-enhancer of activated B cells; TGF-β = Transforming Growth Factor beta; MMP = Matrix Metalloproteinase; TIMP = Tissue Inhibitor of Metalloproteinases; ECM = Extracellular Matrix; SHBG = Sex Hormone–Binding Globulin.

**Table 2:** Vascular/Endothelial Dysfunction, NO/AGEs, and FS.

Domain	Study Type	Core Finding	Implication for FS
Endothelial dysfunction (ED)	Clinical (biomarkers/imaging)	ED associated with LGI, dyslipidemia, oxidative stress; impaired microvascular perfusion.	Local hypoperfusion and abnormal angiogenesis → fibrosis and pain.
Nitric oxide (NO) biology	Experimental/Translational	ADMA, homocysteine, ROS/RNS reduce eNOS activity; peroxynitrite ↑ tissue damage.	Impaired NO signaling favors pro-fibrotic environment.
AGEs–RAGE axis	Clinical (FS biopsies)	Elevated AGEs in FS capsules; RAGE/NF-κB activation confirmed.	AGEs act as active mediators of stiffness and fibrosis.
Lipids/inflamed lipoproteins	Clinical (SR/MA, cohorts)	↑ LDL and total cholesterol in FS; ICAM-1 upregulated in capsule.	Vascular inflammation and fibrosis link.

Note: FS = Frozen Shoulder; ED = Endothelial Dysfunction; LGI = Low-Grade Inflammation; ADMA = Asymmetric Dimethylarginine; ROS = Reactive Oxygen Species; RNS = Reactive Nitrogen Species; eNOS = Endothelial Nitric Oxide Synthase; AGEs = Advanced Glycation End-products; RAGE = Receptor for Advanced Glycation End-products; LDL = Low-Density Lipoprotein; ICAM-1 = Intercellular Adhesion Molecule 1.

**Table 3:** Lifestyle, Psychoneuroimmunology, and Metabolic Drivers.

Domain	Study Type	Core Finding	Implication for FS
Diet & glycemic load	Clinical (SR/MA, cohorts)	HbA1c consistently elevated in FS; high-glycemic/ultra-processed diets → LGI, microbiota dysbiosis.	Diet as modifiable driver; supports anti-inflammatory strategies.
Physical inactivity & sleep	Clinical/Translational	Sedentarism and circadian disruption impair HPA, GH, pain modulation.	Exercise and sleep hygiene key for management.
Psychosocial stress	Clinical (cohorts, RCTs)	Anxiety/depression worsen FS prognosis; psychotherapies improve MSK outcomes.	Supports psychoneuroimmunology-based interventions.
Exposome/EDCs	Experimental/Human observational	BPA, phthalates, cadmium alter ER function and circadian release.	Exposome as overlooked contributor to FS pathogenesis.

Note: FS = Frozen Shoulder; LGI = Low-Grade Inflammation; HbA1c = Glycated Hemoglobin; HPA = Hypothalamic–Pituitary–Adrenal axis; GH = Growth Hormone; RCT = Randomized Controlled Trial; MSK = Musculoskeletal; EDCs = Endocrine-Disrupting Chemicals; BPA = Bisphenol A; ER = Estrogen Receptor.

**Table 4:** Established Evidence vs. Emerging Hypotheses in Frozen Shoulder (FS).

Domain	Established Evidence (Human-Focused)	Emerging Hypotheses (Mechanistic/Early Clinical)	Notes/Level of Evidence
Epidemiology & clinical course	Female predominance; peak in perimenopause/postmenopause; painful capsular stiffness with staged course.	Sex-specific vulnerability linked to estrogen deficiency/resistance; phenotypes differing by metabolic status.	High-quality observational data; sex-hormone linkage under active study.
Histopathology & cytokines	Capsular fibrosis; myofibroblast proliferation; ↑ TGF-β, IL-1β, TNF-α; neoangiogenesis; mast cells.	Alarmins (HMGB1, IL-33, S100A8/9) drive nerve ingrowth and persistent fibrosis.	Multiple biopsy series; alarmin data growing (moderate).
Metabolic biomarkers (glycemia, lipids)	Higher HbA1c and total cholesterol in FS vs. controls (meta-analyses).	AGE–RAGE axis as fibrosis driver; glycation impairs cholesterol traffic → dyslipidemia → hormonal disruption.	Human meta-analyses (strong); mechanistic links plausible (moderate).
Thyroid dysfunction	Hypothyroidism (overt/subclinical) associated with FS; MR studies suggest causal link.	Autoimmune thyroiditis primes chronic inflammatory milieu, amplifying capsular fibrosis.	Systematic reviews & MR (strong); immune amplification (moderate).
Estrogen axis (deficiency/resistance)	Postmenopausal status correlates with worse pain/stiffness; ERs in capsule; estrogen is anti-inflammatory/antifibrotic (indirect clinical support).	Estrogen resistance from LGI/oxidative stress/EDCs; receptor-level interference in capsule tissue.	Human indirect + robust mechanistic data (moderate).
Endothelial dysfunction & NO biology	Endothelial impairment associates with chronic pain states; lifestyle improves endothelial function.	Local microvascular injury in capsule (↓NO/eNOS, ↑ADMA, oxidized LDL) fuels fibrosis & pain.	Human vascular data (moderate); capsule-specific vascular biology emerging (limited–moderate).
Microbiome & gut barrier	Diets high in ultra-processed foods associate with inflammation; gut barrier compromise in chronic pain cohorts.	Dysbiosis → LPS/ADMA → endothelial & ER signaling disruption; gut–joint axis in FS.	Human associative data (moderate); FS-specific data limited.
Psychoneuroimmunology & sleep	Stress, poor sleep linked to worse pain/function; psychoeducation/exercise benefit shoulder pain.	Circadian misalignment → HPA/SNS activation → ER dysfunction and fibrosis.	Human outcomes (moderate); circadian-ER link mechanistic (moderate).
Exposome/EDCs	Population exposure to BPA/phthalates/parabens widespread; endocrine effects documented in humans.	Xenoestrogens cause ER misactivation/desensitization in capsule, promoting fibrosis.	Human exposure strong; FS-targeted evidence limited (emerging).
Adipose tissue dysfunction	Central adiposity associates with systemic inflammation and shoulder pain risk.	Leptin resistance downregulates ERα; adipokines drive fibroblast activation in capsule.	Human association (moderate); cellular mechanisms strong.
Therapeutics—standard care	Exercise therapy, manual therapy, education; injections/hydrodistension as per guidelines.	Stratified care by immunometabolic/endocrine phenotype to personalize response.	High clinical evidence for core PT; precision phenotyping emerging.
Therapeutics—metabolic & endocrine	Diet/exercise improve pain in shoulder disorders; omega-3s analgesic in inflammatory joint pain.	Low-AGE/anti-inflammatory/phytoestrogen diets; circadian therapy; endocrine optimization (thyroid/estrogen) in selected patients.	Mixed clinical evidence (moderate); targeted trials needed.

Note: FS = Frozen Shoulder; ER = Estrogen Receptor; MR = Mendelian randomization; LGI = Low-grade inflammation; AGE = Advanced glycation end-product; RAGE = Receptor for AGE; NO/eNOS = Nitric Oxide/endothelial Nitric Oxide Synthase; ADMA = Asymmetric dimethylarginine; LPS = lipopolysaccharides; HPA = hypothalamic-pituitary-adrenal; SNS = sympathetic nervous system; EDCs = endocrine-disrupting chemicals; PT = physiotherapy.

**Table 5:** Clinical assessment and interventions in Frozen Shoulder (FS) with level of evidence/consensus.

Domain	Clinical Assessments (Recommended)	Interventions (Examples)	Human Clinical Evidence	Experimental/Mechanistic Support
Hormonal—Estrogen/Female axis	Serum estradiol, progesterone, SHBG; menopausal status; vasomotor and sleep symptom scales. (ER polymorphisms for research).	Lifestyle + nutrition to support estrogen balance; phytoestrogens (e.g., soy isoflavones) when appropriate; consider HRT in selected patients per guidelines; vitamin D repletion.	Observational links between menopausal status and FS severity; small trials on phytoestrogens and musculoskeletal symptoms; indirect evidence from shoulder pain cohorts.	Strong evidence of estrogen anti-inflammatory/antifibrotic actions; ER signaling modulates TGF-β/NF-κB; animal & cellular models.
Thyroid axis	TSH, free T4/T3; thyroid autoantibodies (TPOAb/TgAb) if suspected; hypothyroid symptom checklist.	Treat overt/subclinical hypothyroidism per endocrine guidelines; monitor FS outcomes after euthyroid restoration.	Epidemiology, meta-analyses, and Mendelian randomization support association between hypothyroidism and FS.	Thyroid hormones regulate collagen turnover, MMP/TIMP balance, and mitochondrial function in connective tissue.
Metabolic—Glucose/insulin & Lipids	HbA1c, fasting glucose/insulin, HOMA-IR; lipid profile (LDL-C, HDL-C, TG); body composition/waist circumference.	Anti-inflammatory or low-AGE diet; Mediterranean or low-glycemic patterns; weight optimization; structured exercise; omega-3 supplementation as appropriate.	Meta-analyses show elevated HbA1c and cholesterol in FS; clinical trials in shoulder pain show benefit of diet/exercise on pain/function.	AGE-RAGE activation drives fibrosis; hyperglycemia and dyslipidemia impair ECM and endothelial health (cell/animal studies).
Vascular/Endothelial & NO biology	CRP/IL-6 (systemic context); ADMA (research); endothelial function (flow-mediated dilation) in studies; ICAM-1/VCAM-1 (research).	Mediterranean-style diet; homocysteine lowering (B-vitamins) when deficient; antioxidant/mitochondrial support; aerobic exercise.	Emerging human data linking endothelial dysfunction with shoulder pain states; diet/exercise improve endothelial function.	Mechanistic evidence for NO/eNOS impairment, oxidized LDL, and AGE-RAGE in fibrosis and pain sensitization.
Lifestyle & Circadian health	Sleep quality (PSQI), actigraphy if available; chronotype; stress scales; physical activity (IPAQ/accelerometry); dietary quality (MEDAS).	Sleep/circadian hygiene (reduce light-at-night, regular schedule); stress reduction (mindfulness/CBT); graded activity programs; anti-inflammatory nutrition.	Multiple RCTs in musculoskeletal pain show benefit of exercise and psychoeducation; FS RCTs support multimodal rehab; early trials on circadian interventions.	Psychoneuroimmunology models show stress-immune-pain links; animal/experimental data on circadian disruption and inflammation.
Orthopedic/Physiotherapy core care	Pain (NPRS/VAS), SPADI/DASH, ROM (goniometry), strength; functional goals; imaging when indicated.	Progressive exercise therapy, manual therapy, joint mobilization; education & pacing; consider hydrodistension or injections per guidelines.	Strong evidence base for exercise/manual therapy in FS and shoulder disorders; guideline-endorsed multimodal care.	Biomechanical & neurophysiological models support tissue adaptation and central desensitization with rehab.

Note: FS = Frozen Shoulder; SHBG = Sex Hormone–Binding Globulin; ER = Estrogen Receptor; HRT = Hormone Replacement Therapy; TSH = Thyroid-Stimulating Hormone; TPOAb/TgAb = Thyroid Peroxidase/Thyroglobulin Antibodies; HbA1c = Glycated Hemoglobin; HOMA-IR = Homeostatic Model Assessment of Insulin Resistance; LDL-C/HDL-C/TG = Low-/High-Density Lipoprotein Cholesterol/Triglycerides; AGE = Advanced Glycation End-product; RAGE = Receptor for AGE; NO/eNOS = Nitric Oxide/endothelial Nitric Oxide Synthase; CRP = C-reactive Protein; ICAM-1/VCAM-1 = Intercellular/Vascular Cell Adhesion Molecule-1; PSQI = Pittsburgh Sleep Quality Index; IPAQ = International Physical Activity Questionnaire; NPRS/VAS = Numeric Pain Rating Scale/Visual Analog Scale; SPADI/DASH = Shoulder Pain and Disability Index/Disabilities of the Arm, Shoulder and Hand.

## Data Availability

No new data were created or analyzed in this study.

## Supplementary Materials

The following supporting information can be downloaded at: https://www.mdpi.com/article/10.3390/jcm14207315/s1, Table S1: Search strategy, databases, eligibility criteria, and sources by mechanistic domain.
