# Supplementary material for: Frozen Shoulder as a Systemic Immunometabolic Disorder: The Roles of Estrogen, Thyroid Dysfunction, Endothelial Health, Lifestyle, and Clinical Implications
Source: J Clin Med. 2025 Oct 16;14(20):7315. doi: 10.3390/jcm14207315 (PMC12564958; doi:10.3390/jcm14207315)
Supplement: Supplementary file 1 [file jcm-14-07315-s001.zip › jcm-3868250-supplementary.pdf]

Appendix S1 – Table S1. Search strategy, databases, eligibility criteria, and sources by mechanistic domain

Clarification: All domains were searched across the same databases—PubMed, Scopus, and Web of Science—using domain-tailored keywords. Backward/forward citation tracking was applied uniformly. The table below reflects identical database coverage for each domain.

Domain	Databases (uniform across domains)	Search Strategy & Keywords (examples)	Inclusion Criteria	Exclusion Criteria
Estrogen & Thyroid Axes	PubMed; Scopus; Web of Science (Backward/forward citation tracking in all)	"frozen shoulder" OR "adhesive capsulitis" AND (estrogen OR estradiol OR menopause OR estrogen receptor OR progesterone OR thyroid OR hypothyroidism OR hyperthyroidism OR SHBG)	Human observational/interventional studies; systematic/scoping reviews; mechanistic/experimental studies with translational relevance to FS	Opinion-only articles; animal-only studies without human linkage; non-musculoskeletal contexts
Vascular/Endothelial (NO/AGEs)	PubMed; Scopus; Web of Science (Backward/forward citation tracking in all)	"frozen shoulder" OR "adhesive capsulitis" AND (endotheli* OR nitric oxide OR eNOS OR microcirculation OR angiogenesis OR AGEs OR RAGE)	Human studies on endothelial function/biomarkers/imaging in FS; mechanistic studies linking NO/AGEs to fibrosis or inflammation with translational value	Animal-only studies without human linkage; unrelated vascular diseases without FS relevance
Lifestyle, Psychoneuroimmunology & Metabolic Drivers	PubMed; Scopus; Web of Science (Backward/forward citation tracking in all)	"frozen shoulder" OR "adhesive capsulitis" AND (lifestyle OR diet OR ultra-processed OR obesity OR insulin resistance OR dyslipidemia OR stress OR sleep OR circadian OR microbiome OR psychoneuroimmunology)	Human clinical/observational studies; high-quality reviews; mechanistic studies on immune–metabolic pathways relevant to FS	Opinion-only pieces; animal-only studies without human translation; narrative pieces lacking mechanistic or clinical relevance

Note: Last database update: May 2025. No language restrictions were applied at the search level; screening prioritized English and Spanish full texts. Concept saturation guided selection across domains in keeping with the narrative–scoping design.
